# Antigenic characterization of SARS-CoV-2 variants BA.3.2.1 and BA.3.2.2 in three animal models

**DOI:** 10.64898/2026.05.24.727525

**Published:** 2026-05-26

**Authors:** Samuel A. Turner, Joey Olivier, Madison L. Ellis, Katharine A. Floyd, Lilin Lai, Suzanne M. Scheaffer, Ionsei Hastings, Tamarand L. Darling, Blake A. Miller, Charmy J. Patel, Hannah Peck, Daryll Vanover, Philip J. Santangelo, Michael S. Diamond, Mehul S. Suthar, Adrianus C.M. Boon, Derek J. Smith

**Affiliations:** 1 Center for Pathogen Evolution, Department of Zoology, University of Cambridge; Cambridge, CB2 3EJ, UK.; 2 Center for Childhood Infections and Vaccines of Children's Healthcare of Atlanta, Department of Pediatrics, Emory University School of Medicine, Atlanta, Georgia, USA.; 3 Emory Vaccine Center, Emory National Primate Research Center, Emory University, Atlanta, Georgia, USA.; 4 Department of Medicine, Washington University School of Medicine, St. Louis, MO 63110, USA.; 5 Wallace H. Coulter Department of Biomedical Engineering, Georgia Institute of Technology and Emory University, Atlanta, GA 30332, USA.; 6 Department of Pathology and Immunology, Washington University School of Medicine, St. Louis, MO 63110, USA.; 7 Department of Molecular Microbiology, Washington University School of Medicine, St. Louis, MO 63110, USA.

## Abstract

BA.3.2, a variant of SARS-CoV-2 containing ~40 mutations in its spike protein compared to its nearest ancestor, has spread globally since its first detection in South Africa in November 2024. Here, we report antigenic characterization of BA.3.2 viruses in three naive animal models, and visualize its antigenic phenotype in the context of SARS-CoV-2 evolution using antigenic cartography. We find that: (1) BA.3.2 is substantially antigenically divergent from existing SARS-CoV-2 variants; (2) infection with BA.3.2 in hamster and mouse animal models produces sera with lower homologous titer than infection with other variants. Both of these results may have implications for the selection of vaccine antigens.

BA.3.2, a highly mutated variant of SARS-CoV-2, was first detected in South Africa in November 2024^1,2^ and has since spread globally, accounting for ~20% of SARS-CoV-2 sequences worldwide between April and May 2026^3^. Compared to its nearest ancestor, BA.3, the BA.3.2 spike carries 39 amino acid substitutions, two N-terminal domain deletions, and a four-residue insertion — meaning its emergence represents a saltation event similar to those which produced the early Omicron variants and BA.2.86^1,2^. The large number of mutations in the spike protein of BA.3.2 suggests its antigenic phenotype may differ substantially from pre-existing variants.

Here, we report antigenic characterization of BA.3.2 using three naive animal models (see Supplementary methods for details):

1. Mouse serum following two doses of mRNA vaccine.
2. Mouse serum following infection.
3. Hamster serum following infection.

We characterize the two main phylogenetic clades of BA.3.2: BA.3.2.1, which is less prevalent and carries spike substitutions H681R and P1162R; and BA.3.2.2, which is more prevalent and carries spike substitutions K356T and A575S^3^.

We measured FRNT titers (see Supplementary methods for details) for BA.3.2 antigens against a panel of sera raised against historical and contemporary SARS-CoV-2 variants in the mouse mRNA vaccination model (both BA.3.2.1 and BA.3.2.2 antigens) and in the hamster infection model (BA.3.2.2 antigen only) (Figure 1). In the mouse mRNA vaccination model, fold-reduction from the homologous antigen to BA.3.2.1 and BA.3.2.2 antigens was largest for anti-KP.2 sera (>124x to >139x fold-reduction), and smaller both for early Omicron (anti-BA.1 and anti-BA.5) sera (>4.4x to >49x) and anti-XBB.1.5 sera (>10x to >20x). Where detectable, titers against the BA.3.2.1 antigen were nearly always lower than those to the BA.3.2.2 antigen (~2-fold lower on average). In the hamster infection model, titers against the BA.3.2.2 antigen were non-detectable for nearly all sera (D614G, B.1.617.2, BA.1, BA.5, BA.2.86, JN.1, XFG).

In the hamster infection and mouse infection models, we raised sera against the BA.3.2.1 and BA.3.2.2 variants. In both models, homologous titers for these sera were substantially lower than for sera raised to other variants (Figure S1). In the hamster infection model, the majority of titers for anti-BA.3.2.1 sera against the BA.3.2.2 antigen were non-detectable. In contrast, in the mouse infection model, anti-BA.3.2.1 sera had a geometric mean titer of ~160 to the BA.3.2.2 antigen, approximately 2-fold higher than their homologous titer to the BA.3.2.1 antigen. This latter observation indicates potentially higher avidity of the BA.3.2.2 antigen than the BA.3.2.1 antigen, which may also contribute to the titer differences between these antigens in the mouse mRNA vaccination data. The substantial majority of titers for anti-BA.3.2.1 and anti-BA.3.2.2 sera against other variants were non-detectable in the mouse infection model, although titers were detectable against BA.1 and XFG antigens more often than against other antigens.

We visualized the antigenic phenotype of the BA.3.2.1 and BA.3.2.2 variants in the context of historical SARS-CoV-2 variants using antigenic cartography (Figure 2)^4^. In the mouse mRNA vaccination map, BA.3.2.1 and BA.3.2.2 occupy novel antigenic space distinct from any pre-existing variant. Their antigenic positions are located further from JN.1-lineage antigens than from early Omicron (BA.1, BA.2, BA.5) and XBB.1.5 antigens, consistent with the larger fold-reduction observed for anti-KP.2 sera than for early Omicron and XBB.1.5 sera in this model. BA.3.2-lineage and JN.1-lineage antigens are approximately equidistant from pre-Omicron and early Omicron antigens. This distance matters because most individuals' first SARS-CoV-2 exposures were to pre-Omicron or early Omicron variants — often in the form of highly immunogenic mRNA vaccines — resulting in substantial immune imprinting on those variants^5–8^. The comparable antigenic distances from these antigens to BA.3.2 and to JN.1-lineage variants are therefore consistent with observations in multiple human cohorts of similar neutralizing titer to BA.3.2-lineage and JN.1-lineage variants^9–11^.

Due to the small number of detectable titers for BA.3.2.1 and BA.3.2.2 antigens and sera in the hamster and mouse infection models, it was not possible to resolve the antigenic position of the variants in these maps.

These data indicate that BA.3.2 is substantially antigenically divergent from any existing SARS-CoV-2 variant. As such, despite the relatively modest reduction in human serum titers from the currently circulating JN.1-lineage variants (XFG and NB.1.8.1) to BA.3.2, BA.3.2 is nonetheless substantially antigenically distinct. This is consistent with observations that vaccination with a JN.1-lineage variant only modestly boosts BA.3.2 titers^9–11^, and suggests the boost to JN.1-lineage titers from a BA.3.2 vaccination may be similarly modest. The observation of low homologous titer for anti-BA.3.2 sera in the hamster and mouse infection models may also be relevant when considering the potential immunogenicity of a BA.3.2 vaccine antigen.

## Supplementary Material

Supplement 1

## Figures and Tables

**Figure 1.**
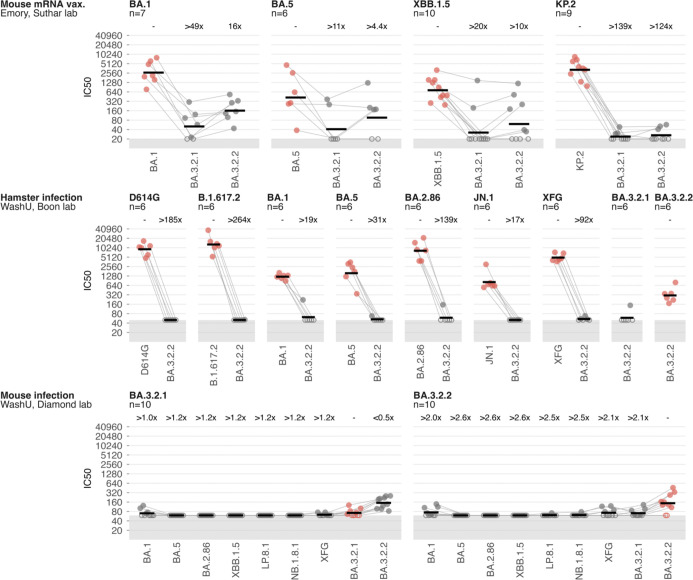
Neutralization titers against BA.3.2.1 and BA.3.2.2 antigens compared to historical and contemporary variants. Each panel shows sera raised against the antigen named in the panel header. Different rows show data from three different animal models. Red points show titers against the homologous antigen. Open circles show titers at or below the limit of detection (LOD). Black horizontal bars show the geometric mean titer, treating titers below the LOD as equal to the LOD. The geometric mean fold-reduction relative to the homologous antigen is written above each heterologous antigen. “>“ and “<“ indicate lower and upper bounds on the fold-reduction in cases where some titers were below the limit of detection.

**Figure 2.**
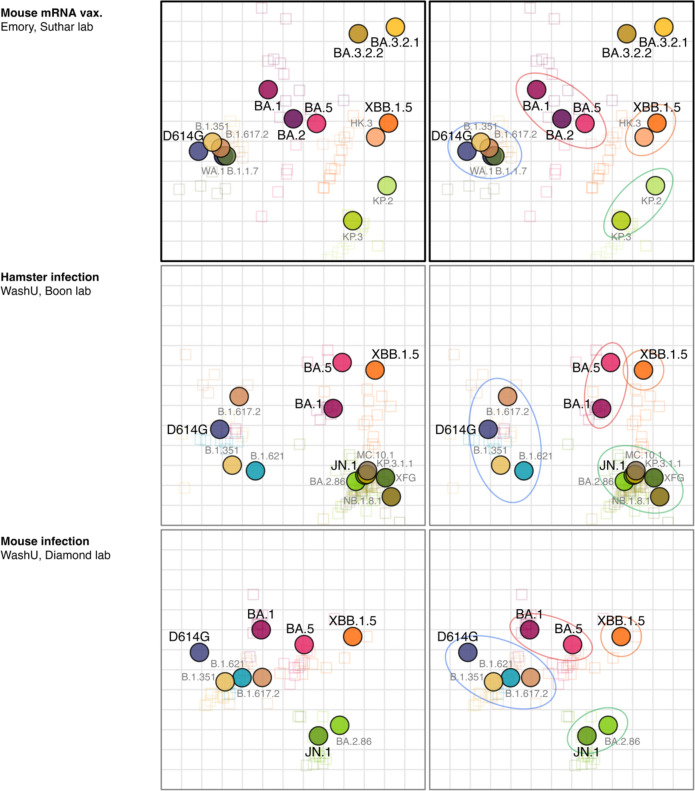
Antigenic maps for the three serum panels. Each row shows a map from one animal model, listed in the left column. Ellipses in the right-hand maps show the major antigenic groups: ancestral lineages, blue; early Omicron, red; XBB lineage, orange; JN.1 lineage, green. Points are antigens, colored by variant; open squares are individual sera. One grid square represents one antigenic unit, corresponding to a two-fold change in titer. All maps are Procrustes-aligned to the mRNA vaccination map (Emory, Suthar). BA.3.2.1 and BA.3.2.2 antigens and sera are not plotted in the hamster and mouse infection maps, as they could not be positioned due to the small number of detectable titers for these variants in these models.
